# Application of Indian Diabetic Risk Score (IDRS) and Community Based Assessment Checklist (CBAC) as Metabolic Syndrome prediction tools

**DOI:** 10.1371/journal.pone.0283263

**Published:** 2023-03-27

**Authors:** Manoj Kumar Gupta, Gitashree Dutta, Sridevi G., Pankaja Raghav, Akhil Dhanesh Goel, Pankaj Bhardwaj, Suman Saurabh, Srikanth S., Naveen K. H., Prasanna T., Neeti Rustagi, Prem Prakash Sharma

**Affiliations:** 1 Department of Community Medicine & Family Medicine, All India Institute of Medical Sciences (AIIMS), Jodhpur, Rajasthan, India; 2 School of Public Health (SPH), All India Institute of Medical Sciences (AIIMS), Jodhpur, Rajasthan, India; UT Southwestern: The University of Texas Southwestern Medical Center, UNITED STATES

## Abstract

**Background:**

Indian Diabetic Risk Score (IDRS) and Community Based Assessment Checklist (CBAC) are easy, inexpensive, and non-invasive tools that can be used to screen people for Metabolic Syndrome (Met S). The study aimed to explore the prediction abilities of IDRS and CBAC tools for Met S.

**Methods:**

All the people of age ≥30 years attending the selected rural health centers were screened for Met S. We used the International Diabetes Federation (IDF) criteria to diagnose the Met S. ROC curves were plotted by taking Met S as dependent variables, and IDRS and CBAC scores as independent/prediction variables. Sensitivity (SN), specificity (SP), Positive and Negative Predictive Value (PPV and NPV), Likelihood Ratio for positive and negative tests (LR^+^ and LR^-^), Accuracy, and Youden’s index were calculated for different IDRS and CBAC scores cut-offs. Data were analyzed using SPSS v.23 and MedCalc v.20.111.

**Results:**

A total of 942 participants underwent the screening process. Out of them, 59 (6.4%, 95% CI: 4.90–8.12) were found to have Met S. Area Under the Curve (AUC) for IDRS in predicting Met S was 0.73 (95%CI: 0.67–0.79), with 76.3% (64.0%-85.3%) sensitivity and 54.6% (51.2%-57.8%) specificity at the cut-off of ≥60. For the CBAC score, AUC was 0.73 (95%CI: 0.66–0.79), with 84.7% (73.5%-91.7%) sensitivity and 48.8% (45.5%-52.1%) specificity at the cut-off of ≥4 (Youden’s Index, 2.1). The AUCs of both parameters (IDRS and CBAC scores) were statistically significant. There was no significant difference (p = 0.833) in the AUCs of IDRS and CBAC [Difference between AUC = 0.00571].

**Conclusion:**

The current study provides scientific evidence that both IDRS and CBAC have almost 73% prediction ability for Met S. Though CBAC holds relatively greater sensitivity (84.7%) than IDRS (76.3%), the difference in prediction abilities is not statistically significant. The prediction abilities of IDRS and CBAC found in this study are inadequate to qualify as Met S screening tools.

## Introduction

An accumulation of clinical, physiological, biochemical, and metabolic factors which increases the risk of cardiovascular disease, type 2 diabetes mellitus (DM), and all-cause mortality is known as Metabolic Syndrome (Met S) [1]. Rapid urbanization, globalization, and the adoption of unhealthy lifestyles, such as unhealthy dietary habits and lack of physical activity, are vital factors in the development of major Non-Communicable Diseases (NCDs), which can manifest as hypertension, diabetes, hyperlipidemia, and obesity. These metabolic risk factors can be modified and controlled if detected early [2, 3].

Due to the unique "atherogenic dyslipidemic profile" and "South Asian phenotype," Indians have a significant chance of developing Met S [4, 5]. The incidence of Met S often parallels the incidences of obesity and type 2 diabetes [11]. Met S also increases the risk of type 2 diabetes and cardiovascular illnesses, including stroke and myocardial infarction, by five and twofold in 5 to 10 years [12, 13]. The Global Burden of Disease (GBD) study revealed the presence of epidemiological transition in India, where 62.7% of the total mortality was attributed to NCDs [6].

Though there are differences in diagnostic criteria of Met S, and the published evidence also varies with respect to the age of study participants and methodology, a significant epidemic of Met S is emerging in the Asia-Pacific region [7]. According to a systematic review of Indian studies, the overall pooled prevalence of Met S among the adult population was 30% (95%CI: 28%-33%) [8]. The secondary data analysis of the fourth round of the National Family Health Survey (NFHS-4) presented that the prevalence of Met S was higher among women than men in India. The data also revealed that the chance of developing Met S increases with age [9].

In a country like India, the western world’s influence on dietary patterns, which replace fiber-rich foods with refined carbohydrates and saturated fats, has a significant impact on the expanding obesity epidemic which in turn increases the burden of Met S. A significant association between anthropometric risk factors and Met S among Indian adults has been documented by a systematic review of observational studies which highlighted that people with overweight (pooled OR, 5.47; 95% CI, 3.70–8.09) or obesity (pooled OR, 5.00; 95% CI, 3.61–6.93) had higher odds of having Met S than those of normal or low body weight [10]. Due to the rising burden of obesity, Met S has also emerged as a public health issue among children and adolescents, especially in low and middle-income countries [13].

The Met S is driving the twin global epidemics of type 2 diabetes and CVD. There is an enormous moral, medical, and economic responsibility to identify persons with Met S early so that lifestyle modifications and treatment may avoid the development of diabetes and cardiovascular disease.

To prevent and control major NCDs, the Government of India started the National Programme for Prevention & Control of Cancer, Diabetes, Cardiovascular Diseases & Stroke (NPCDCS) in 2010. Grass-root workers use a Community-Based Assessment Checklist (CBAC) under this program. This checklist is based on two non-modifiable (age and family history of NCDs) and four modifiable (waist circumference, smoking, alcohol, and physical inactivity) known risk factors of NCDs. This checklist helps in screening high-risk individuals for NCDs [11]. The Madras Diabetes Research Foundation (MDRF) in Chennai has developed the Indian Diabetes Risk Score (IDRS), a simple and cost-effective diabetes screening tool based on two modifiable (waist circumference and physical inactivity) and two non-modifiable (age and family history of diabetes) risk factors [12, 13].

There are no screening standards or procedures in place in India for Met S. These (IDRS and CBAC) easy and inexpensive tools, which have already been scientifically validated and are widely used to screen patients for NCDs, can also be used to screen people for Met S in the country. Before this, it is necessary to assess those tools’ prediction abilities (sensitivity and specificity). So, the current study was conducted to screen persons for Met S who visited primary health centers and to explore the prediction abilities of IDRS and CBAC tools for Met S.

## Material and methods

This facility-based cross-sectional study was carried out from January to December 2019 in the three Rural Health Training Centres (RHTCs) of the Department of Community Medicine and Family Medicine at a tertiary care hospital in Jodhpur, India. Out of these three centers, one is Community Health Centre (CHC), and the other two are Primary Health Centres (PHCs). The three health centers’ catchment areas serve approximately one lakh sixty thousand population. All the patients of age ≥ 30 years attending these health centers for various ailments were included in the study. Pregnant females and patients already diagnosed with cholesterol gallstones, asthma, Polycystic ovary syndrome (PCOS), sleep apnoea, autoimmune disorders, and cancer of colorectal (colon and rectum), gastric, oesophageal, hepatobiliary (liver and gallbladder), pancreas, lung, bladder, thyroid, renal, leukaemia, malignant melanoma, multiple myeloma, and non-Hodgkin lymphoma were excluded from the study.

The detailed methodology of the study is mentioned in another published article [14].

For operational purposes, the subjective assessments about addictions and physical activity were made based on IDRS and CBAC forms. As per the CBAC form, a drinker is a person who consumes alcohol daily, irrespective of quantity. A smoker was categorized as one who used to smoke in the past/sometimes now and another who smokes on a daily basis. According to CBAC, physical activity was defined as any dedicated physical activity for a minimum of 150 minutes in a week. While in IDRS, regular exercise and strenuous work were considered separately. The presence of a family history (any one of the parents or siblings) of high blood pressure, diabetes, and heart disease was considered a positive family history for NCDs, as per CBAC. While in IDRS, different scores were assigned to having a positive family history of only diabetes in either parent or both parents.

We used the International Diabetes Federation (IDF) criteria to diagnose Met S. Central obesity (defined by the waist circumference with ≥90 cm for males and ≥80 cm for females) was the mandatory criterion. If BMI was >30kg/m^2^, central obesity was assumed irrespective of the waist circumference. The other two criteria used were Blood Pressure (systolic BP ≥ 130 or diastolic BP ≥ 85 mm Hg) and Raised fasting plasma glucose (FPG ≥ 100 mg/dL) [15].

ROC curves were plotted using Met S as dependent variables and IDRS and CBAC scores as independent/prediction variables. Sensitivity (SN), specificity (SP), Positive Predictive Value (PPV), Negative Predictive Value (NPV), Likelihood Ratio for positive test (LR^+^), Likelihood Ratio for negative test (LR^-^)Accuracy, and Youden’s index were calculated for different cut-offs of IDRS and CBAC scores.

The study was approved by the Institutional Ethics Committee of All India Institute of Medical Sciences, Jodhpur (Letter No. AIIMS/IEC/2018/429 dated 19-03-2018). Informed written consent was obtained from all the study participants. Individuals diagnosed with diabetes, hypertension, and Met S were started on treatment and referred to tertiary care hospitals to screen for complications. These patients were given comprehensive counseling about their disease, its complication, and the necessary lifestyle and dietary modification required. Pre-diabetics and pre-hypertensive were explained their risk of developing diabetes or hypertension and were recommended frequent follow-ups.

## Results

Out of the total 984 participants who were eligible for the screening, 942 consented to undergo the screening process (non-consent rate; 4.2%). The mean age of the participants was 52.3 (SD: 13.5) years and ranged from 30 to 94 years. There were 469 (49.8%) males and 473 (50.2%) females. 629 (66.8%) participants performed regular physical activity. History of smoking and opium consumption was reported by 34.0% and 11.3% of participants, respectively **(Table 1)**.

**Table 1:** Sociodemographic and behavioral characteristics of the study participants (n = 942).

Variable	n (%)
**Age (years) (Mean: 52.3±13.5)**	
≤40	266 (28.2)
41–50	205 (21.8)
51–60	196 (20.8)
>60	275 (29.2)
**Gender**	
Male	469 (49.8)
Female	473 (50.2)
**Physical activity**	
Regular	629 (66.8)
No/Minimal physical activity	313 (33.2)
**Addiction**	
Smoking	320 (34.0)
Alcohol	27 (2.9)
Opium	106 (11.3)

**Fig 1:**
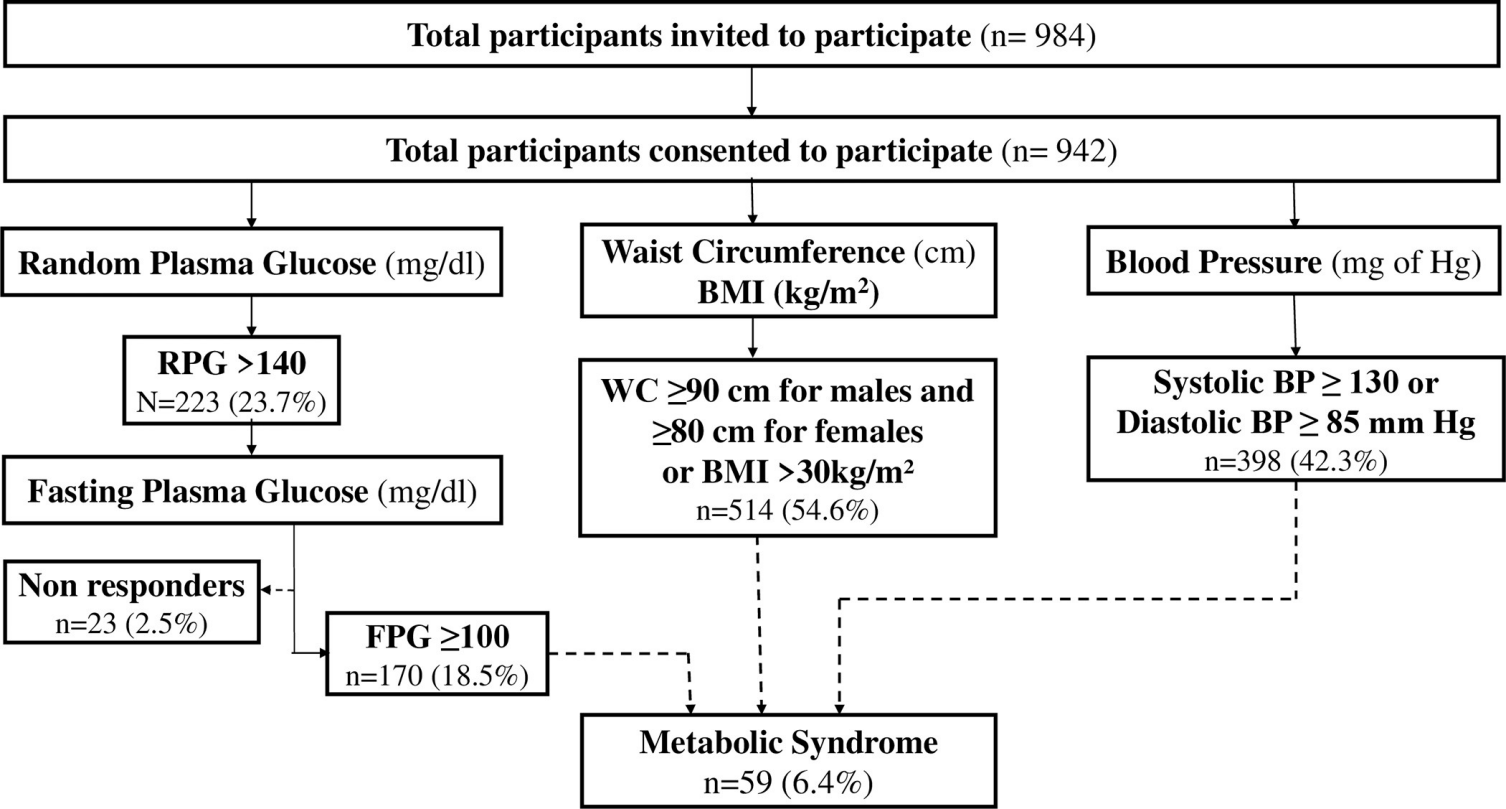
Flow chart depicting screening of the study participants for Metabolic Syndrome.

Of these 942 participants, 514 (54.6%) were found with central obesity as per the criteria mentioned above. A total of 223 (23.7%) were identified as screen positives (RPG ≥140) for diabetes and were invited to undergo FPG. Out of them, 200 (response rate 89.6%) reported undergoing FPG. Despite taking measures to counsel the participants to come for the follow-up to screen for the presence of diabetes, 23 participants did not respond. After excluding non-responders, the proportion of participants with FPG ≥100 mg/dl was 18.5% (170). All the participants (942) were screened for Hypertension, and 42.3% were found with either systolic BP ≥ 130 or diastolic BP ≥ 85 mm Hg. A total of 59 participants (6.4%, 95% CI: 4.90–8.12) were found to have Met S **(Fig 1)**.

**Fig 2:**
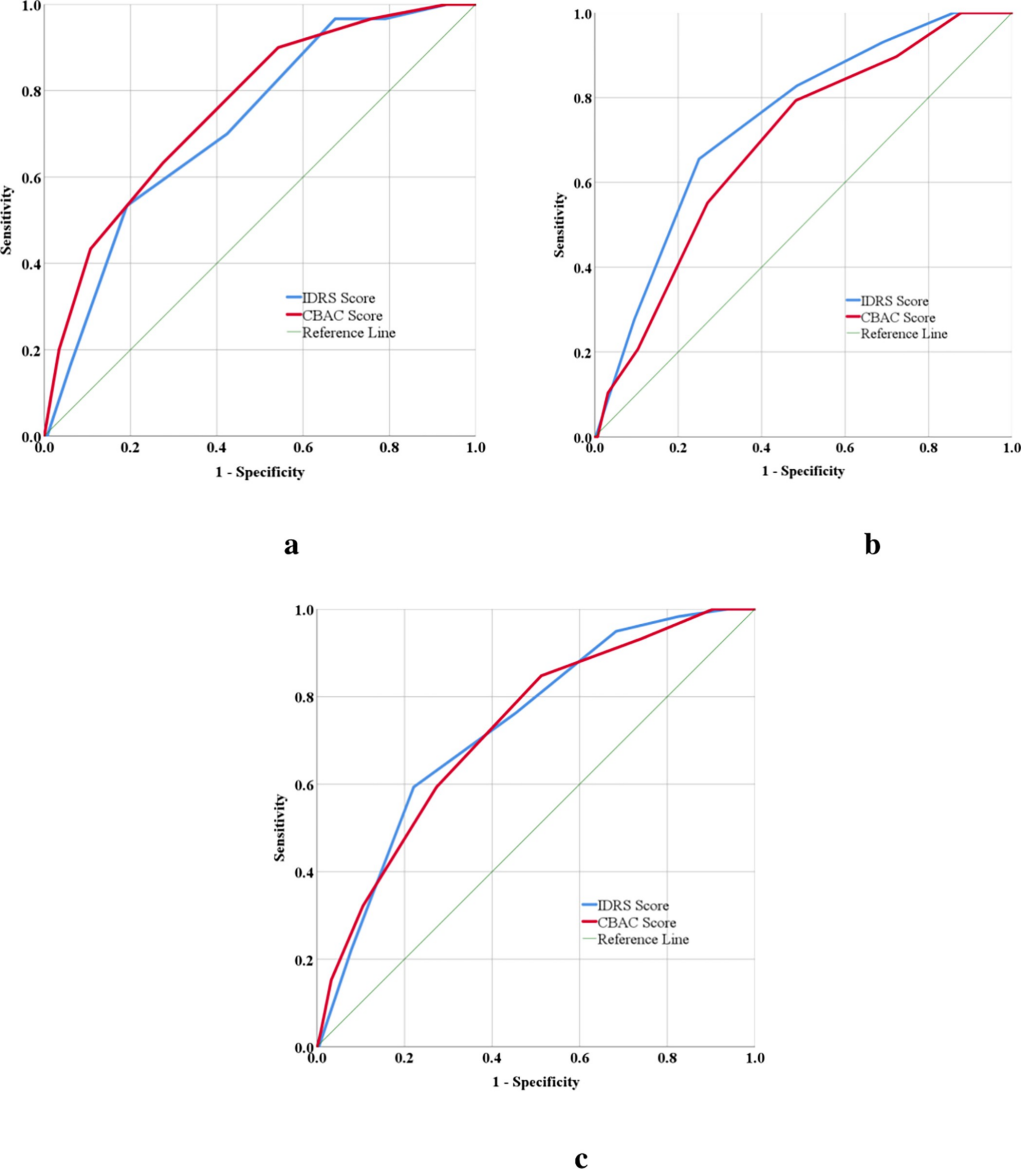
ROC curves for depicting prediction ability of Met S with IDRS and CBAC score for Metabolic Syndrome (2a: ROC curves for Males, 2b: ROC curves for Females, 2c: ROC curves for total participants).

Cut-off points of IDRS and CBAC scores were estimated using ROC curves for Met S **(Fig 2)**.

On applying univariate analysis, it was observed that age (p = 0.120) and sex (p = 0.866) were not significantly associated with Met S. The Area Under the Curve (AUC) and level of significance for ROC curves for male, female and total are depicted in **Table 2**. AUC of both the parameters (IDRS and CBAC scores) was found to be statistically significant.

**Table 2:** Area Under the Curve (AUC) and level of significance for ROC curves.

Variables	Metabolic Syndrome					
	Male		Female		Total	
	AUC (95% CI)	Sig.	AUC (95% CI)	Sig.	AUC (95% CI)	Sig.
IDRS	0.72 (0.64–0.81)	<0.001	0.74 (0.66–0.83)	<0.001	0.73 (0.67–0.79)	<0.001
CBAC	0.76 (0.68–0.84)	<0.001	0.69 (0.60–0.78)	0.001	0.73 (0.66–0.79)	<0.001

On further analysis, it was noted that there was no significant difference (p = 0.833) in the AUCs of IDRS and CBAC [Difference between AUC = 0.00571] **(Table 3)**.

**Table 3:** Statistical comparison of all the ROC curves of IDRS and CBAC scores.

Variables	Difference b/w AUCs	SE* of difference	p value
IDRS (Total) v/s CBAC (Total)	0.005	0.0541	0.833
IDRS (Male) v/s CBAC (Male)	0.039	0.0749	0.602
IDRS (Female) v/s CBAC (Female)	0.053	0.0776	0.494
IDRS (Male) v/s IDRS (Female)	0.022	0.0762	0.772
CBAC (Male) v/s CBAC (Female)	0.071	0.0764	0.359

*SE- Standard Error.

**Table 4** provides the sensitivity, specificity, PPV, NPV, **LR**^**+**^**, LR**^**-**^, and accuracy of different cut-offs for IDRS and CBAC scores for predicting Met S. An IDRS value ≥60 and CBAC value of ≥4 had the optimum sensitivity (76.3% and 53.3%, respectively) and specificity (84.7% and 48.8%, respectively) for determining Met S.

**Table 4:** Sensitivity, specificity, PPV, NPV, LR^+^, LR^-^, accuracy, and Youden’s index of IDRS and CBAC scores to diagnose Met S.

Score	SN, %(95%CI)	SP, %(95%CI)	PPV (%)	NPV (%)	LR^+^ (95%CI)	LR^-^ (95%CI)	Accuracy (%)	Youden’s index
**IDRS Score**								
≥ 10	100 (93.9–100)	1.4 (0.8–2.4)	6.3	100.0	1.01 (1.01–1.02)	0.00	7.5	1.4
≥ 20	100 (93.9–100)	2.2 (1.4–3.3)	6.4	100.0	1.02 (1.01–1.03)	0.00	8.3	2.2
≥ 30	100 (93.9–100)	6.1 (4.7–7.9)	6.6	100.0	1.07 (1.05–1.08)	0.00	12.0	6.1
≥ 40	98.3 (91.0–99.7)	17.4 (15.1–20.1)	7.4	99.4	1.19 (1.14–1.25)	0.10 (0.01–0.68)	22.5	15.7
≥ 50	94.9 (86.1–98.3)	31.7 (28.7–34.8)	8.5	98.9	1.39 (1.29–1.50)	0.16 (0.05–0.48)	35.7	26.6
≥ 60	76.3 (64.0–85.3)	54.6 (51.2–57.8)	10.1	97.2	1.68 (1.43–1.97)	0.43 (0.27–0.69)	55.9	30.9
≥ 70	59.3 (46.6–70.9)	77.9 (75.9–81.4)	15.2	96.6	2.69 (2.10–3.43)	0.52 (0.38–0.71)	76.8	37.2
≥ 80	22.0 (13.3–34.1)	92.2 (90.2–93.8)	15.9	94.7	2.82 (1.66–4.79)	0.85 (0.74–0.97)	87.8	14.2
≥ 90	0.0 (0.0–6.1)	99.5 (98.8–99.8)	0.0	93.7	0.00	1.00 (1.00–1.00)	93.3	0.0
**CBAC Score**								
≥ 1	100 (93.9–100)	2.6 (1.7–3.9)	6.4	100.0	1.03 (1.02–1.04)	0.00	8.7	2.6
≥ 2	100 (93.9–100)	9.5 (7.7–11.6)	6.9	100.0	1.11 (1.08–1.13)	0.00	15.2	9.5
≥ 3	93.2 (83.8–97.3)	25.9 (23.1–28.9)	7.8	98.3	1.26 (1.16–1.36)	0.26 (0.10–0.68)	30.1	1.2
≥ 4	84.7 (73.5–91.7)	48.8 (45.5–52.1)	10.0	98.0	1.66 (1.46–1.88)	0.31 (0.17–0.57)	51.1	2.1
≥ 5	59.3 (46.6–70.9)	72.7 (69.7–75.5)	12.7	96.4	2.17 (1.71–2.76)	0.56 (0.41–0.76)	71.9	2.0
≥ 6	32.2 (21.7–44.9)	89.5 (87.3–91.3)	17.0	95.2	3.06 (2.01–4.64)	0.76 (0.63–0.90)	85.9	1.4
≥ 7	15.3 (8.2–26.5)	96.7 (95.3–97.7)	23.7	94.5	4.64 (2.31–9.35)	0.88 (0.79–0.98)	91.6	12.0
≥ 8	3.4 (0.9–11.5)	99.1 (98.2–99.5)	20.0	93.9	3.74 (0.81–17.23)	0.97 (0.93–1.02)	93.1	2.5
≥ 9	0.0 (0.0–6.1)	99.9 (99.3–99.9)	0.0	93.7	0.00 (0.00–0.00)	1.00 (1.00–1.00)	93.6	0.0

## Discussion

In this study, facility-based screening of rural people of Western Rajasthan was done to estimate the proportion of newly diagnosed cases of Met S. The prediction capacity of the existing non-invasive IDRS and CBAC scores was calibrated to screen for Met S, and the appropriate cut-offs were determined.

Met S was diagnosed among 6.4% of participants in the present study. This is relatively lower than the prevalence (15.6%) reported by a large survey in India among the rural adult population [16]. A study from Central India has reported a 5% prevalence of Met S in the rural adult population [17]. Another study from South India has reported 39.7% (95% CI: 35.3–44.1) prevalence among the rural adult population [18]. According to the secondary data analysis of NFHS-4, the prevalence of Met S was 1.5% among women and 1.1% among men in India. While in Rajasthan, it was 0.8% (0.536–0.966) among women and 1.0 (0.859–1.052) among men [9]. This varied prevalence across the different geographical regions of the country may be because of using different criteria for diagnosing Met S or having different age cut-offs of the study populations.

Though IDRS is used to screen for diabetes, we tried to explore its prediction ability to diagnose Met S in this study. In our study, AUC for IDRS score in predicting Met S was 0.73 (95%CI: 0.67–0.79), with 76.3% sensitivity and 54.6% specificity at the cut-off of ≥60, and with 59.3% sensitivity and 77.9% specificity at the cut-off of ≥70. When the major part of the disease iceberg is hidden in the community, and untreated disease could end up with serious complications, a score that maximizes true positives is preferable. So, the screening cut-off is often set at a lower level, which increases the sensitivity value. Thus, IDRS with a cut-off of ≥60 is more appropriate to predict Met S. The present study’s findings are well supported by a large study conducted in the Southern part of India [19]. The study by Mohan V. et al. (2013) also concluded that IDRS could help to identify Met S by observing the increased prevalence of Met S among those with high IDRS Scores [20]. But, the prediction ability of IDRS found in this study is inadequate to qualify as a Met S screening tool.

The results of this study also show the prediction ability of the CBAC form, which is extensively used in India by front-line healthcare workers to screen for NCDs. Traditionally, in the screening using the CBAC checklist, a score above 4 indicates that the person may be at risk for NCDs. In our study, AUC for CBAC score in predicting Met S was 0.73 (95%CI: 0.66–0.79), with 84.7% sensitivity and 48.8% specificity at the cut-off of ≥4 (Youden’s Index, 2.1). There is a dearth of scientific evidence exploring the prediction capacity of CBAC form for Met S. Based on the findings of this study; the CBAC tool also does not seem promising in identifying people at high risk of developing Met S in primary care settings.

The most accurate metric for gauging the effectiveness of a risk score is the total area under the ROC curve. The risk score performs better overall in terms of adequately predicting persons who will get a disease when the area under the curve is higher [21]. The present study depicts that the AUC of IDRS and CBAC scores for predicting Met S were almost similar (0.731 and 0.726, respectively). Based on the detection of true positives (sensitivity) also, IDRS at the cut-off score of ≥60 has slightly lower values (76.3%) than the CBAC checklist at the cut-off of ≥4 (84.7%). There was no significant difference between the AUCs of IDRS and CBAC scores. This slight difference in the AUCs between IDRS and CBAC can be attributed to the appropriate giving consideration to tobacco and alcohol use in the CBAC checklist compared to the IDRS, which gives a substantially higher priority to factors including age, family history, physical activity, and central obesity.

This study was non-funded, so the HDL and Triglyceride levels of the study participants could not be assessed. This may underestimate the prevalence of Met S in the study. This is one of the limitations of this study. Besides that, the authors did not collect data related to the diet of study participants, so the effect of diet could not be adjusted for the prediction of Met S using IDRS and CBAC.

## Conclusion and recommendations

The current study provides scientific evidence that both IDRS and CBAC have almost 73% prediction ability for Met S. Though CBAC holds relatively greater sensitivity (84.7%) than IDRS (76.3%), the difference in prediction abilities is not statistically significant. The prediction abilities of IDRS and CBAC found in this study are inadequate to qualify as Met S screening tools. Thus, both tools do not seem promising in identifying people at high risk of developing Met S in primary care settings. As these tools are the best available non-invasive tools for screening NCDs and are widely used in India, their prediction abilities need to be explored further with a larger sample size and multicentric basis by considering the limitations mentioned in this study.

## Supporting information

S1 File Data sheet. (XLSX)
